# Associations between COPD related manifestations: a cross-sectional study

**DOI:** 10.1186/1465-9921-14-129

**Published:** 2013-11-19

**Authors:** Elisabeth APM Romme, David A McAllister, John T Murchison, Edwin JR Van Beek, George S Petrides, Cameron OS Price, Erica PA Rutten, Frank WJM Smeenk, Emiel FM Wouters, William MacNee

**Affiliations:** 1 Department of Respiratory Medicine, Catharina Hospital, Eindhoven, The Netherlands; 2 Department of Respiratory Medicine, Maastricht University Medical Centre + (MUMC+), P.O. Box 5800, Maastricht 6202 AZ, The Netherlands; 3 Centre for Population Health Sciences, University of Edinburgh, Edinburgh, Scotland, UK; 4 Department of Radiology, Royal Infirmary of Edinburgh, Edinburgh, Scotland, UK; 5 Clinical Research Imaging Centre, University of Edinburgh, Edinburgh, Scotland, UK; 6 Department of Radiology, Freeman Hospital, Newcastle, UK; 7 Program Development Centre, Centre of expertise for chronic organ failure + (CIRO+), Horn, The Netherlands; 8 Centre for Inflammation Research, University of Edinburgh, Edinburgh, Scotland, UK

**Keywords:** Arterial calcification, Arterial stiffness, Bone density, Cardiovascular disease, Co-morbidity, Computed tomography, COPD, Emphysema, Mortality, Osteoporosis

## Abstract

**Background:**

Cardiovascular disease, osteoporosis and emphysema are associated with COPD. Associations between these factors and whether they predict all-cause mortality in COPD patients are not well understood. Therefore, we examined associations between markers of cardiovascular disease (coronary artery calcification [CAC], thoracic aortic calcification [TAC] and arterial stiffness), bone density (bone attenuation of the thoracic vertebrae), emphysema (PI-950 and 15th percentile) and all-cause mortality in a COPD cohort.

**Methods:**

We assessed CAC, TAC, bone attenuation of the thoracic vertebrae, PI-950 and 15th percentile on low-dose chest computed tomography in COPD subjects. We measured arterial stiffness as carotid-radial pulse wave velocity (PWV), and identified deaths from the national register.

**Results:**

We studied 119 COPD subjects; aged 67.8 ±7.3, 66% were males and mean FEV_1_% predicted was 46.0 ±17.5. Subjects were classified into three pre-specificed groups: CAC = 0 (n = 14), 0 < CAC ≤ 400 (n = 41) and CAC > 400 (n = 64). Subjects with higher CAC were more likely to be older (p < 0.001) and male (p = 0.03), and more likely to have higher systolic blood pressure (p = 0.001) and a history of hypertension (p = 0.002) or ischemic heart disease (p = 0.003). Higher CAC was associated with higher PWV (OR 1.62, p = 0.04) and lower bone attenuation (OR 0.32, p = 0.02), but not with 15th percentile, after adjustment for age, sex and pack-years of smoking. In a Cox proportional hazards model, CAC, TAC and 15th percentile predicted all-cause mortality (HR 2.01, 2.09 and 0.66, respectively).

**Conclusions:**

Increased CAC was associated with increased arterial stiffness and lower bone density in a COPD cohort. In addition, CAC, TAC and extent of emphysema predicted all-cause mortality.

**Trial registration:**

Lothian NHS Board, Lothian Research Ethics Committee, LREC/2003/8/28.

## Background

Chronic obstructive pulmonary disease (COPD), in addition to its pulmonary manifestations, is associated with significant extrapulmonary manifestations. Cardiovascular disease and osteoporosis are recognised extrapulmonary manifestations, whose prevalence is higher in COPD patients than in control subjects matched for age and sex [1,2].

In general population studies, a relationship has been suggested between cardiovascular disease and osteoporosis. Postmenopausal women with osteoporosis had a 3.9-fold increased risk of cardiovascular events compared with postmenopausal women with osteopenia [3]. In 2348 postmenopausal women, increased aortic calcification, a marker of cardiovascular disease, was associated with lower bone density and an increased number of vertebral and hip fractures [4], and in a subpopulation studied longitudinally, progression of aortic calcification was associated with bone loss [4].

Although cardiovascular disease and osteoporosis are common in COPD patients [1,2], there are few studies on the relationship between cardiovascular disease and osteoporosis in COPD. Sabit and colleagues [5] found that COPD subjects with osteoporosis had increased arterial stiffness, another marker of cardiovascular disease, compared with COPD subjects without osteoporosis. In addition, arterial stiffness and osteoporosis have been shown to relate to the extent of emphysema [6,7]. These data suggest associations between cardiovascular disease, osteoporosis and emphysema. Furthermore, COPD related extrapulmonary manifestations are thought to contribute to morbidity and mortality [8].

The objectives of this study were: 1. To determine the associations between markers of cardiovascular disease (coronary artery calcium [CAC], thoracic aortic calcium [TAC] and arterial stiffness), bone attenuation of the thoracic vertebrae, and extent of emphysema, and 2. To identify whether these factors predict all-cause mortality in a COPD cohort.

## Methods

Our analysis was based on the data of a cohort study designed to identify prognostic markers in COPD [6]. The study was conducted in accordance with the amended Declaration of Helsinki, the protocol was approved by the Lothian Research Ethics Committee (LREC/2003/8/28), and all subjects gave written informed consent.

### Subjects

The inclusion and exclusion criteria have been described previously [6]. In summary, all subjects were included between April 2003 and December 2005, had a clinical history compatible with COPD, a history of smoking for at least 10 pack-years and evidence of chronic airflow limitation on spirometry. All subjects who had low-dose computed tomography (CT) of the chest were included in our analysis.

Height, weight and post-bronchodilator spirometry were measured according to American Thoracic Society/European Respiratory Society standards. The subjects’ self-reported respiratory symptoms, medications, smoking history, occupational exposure, and coexisting medical conditions were documented at study entry using structured interviews.

### Arterial stiffness

Peripheral blood pressure was measured in all subjects, and arterial stiffness was assessed in 104 subjects. Arterial stiffness was measured as carotid-radial pulse wave velocity (PWV) as described previously [6]. Briefly, we used the Q-wave of a simultaneously recorded electrocardiograph to identify the onset of the pressure wave, and used applanation tonometry of the carotid and radial arteries to record the pressure waveform at the peripheral site. The difference in wave transit time between the carotid and radial arteries was used to calculate carotid-radial PWV.

### CT scanning

Low-dose CT scanning of the chest was performed without contrast media at full inspiration using a 16-slice multi-detector-row CT scanner (135 kV, 20 mAS; Toshiba Aquilion, Toshiba, Japan). Images were reconstructed with a slice thickness of 1 mm increment using an FC-03 filter (Toshiba, Japan).

CAC and TAC were assessed on the standard images used for analysis of the lungs as previously described [9]. Images were analysed on a dedicated post-processing workstation (VOXAR 3D) using calcium analysis software. CAC and TAC were quantified using the Agatston score [10]. Calcification was defined as an area ≥1 mm^2^ in the axial plane of a coronary artery or the thoracic aorta with an attenuation threshold of ≥130 Hounsfield units. Regions of interest were drawn, and CAC and TAC were calculated by multiplying by a weighting factor selected dependent on the peak signal within the region of interest. Total CAC was obtained by summing the weighted scores from each coronary artery, and total TAC was obtained by summing the weighted scores from the ascending aorta, aortic arch and descending aorta superiorly to the upper limit of the thoracic vertebrae 12 (T12). The CT images of a random sample of 25 patients were independently assessed by two observers and assessed by one observer twice. The intra-class correlation coefficients were high for inter-observer and intra-observer agreements for CAC (0.88 and 0.99 respectively) and TAC (both 0.99).

Emphysema was quantified by in-house software using the 15th percentile point of the frequency distribution of lung attenuation, and pixel index for -950 (PI-950) Hounsfield units as previously described [6]. Bone attenuation of the thoracic vertebrae was measured according to a method described in detail previously [11]. The mean bone attenuation of thoracic vertebrae 4, 7 and 10 (T4, T7 and T10) were determined by placing circular regions of interest in the central parts of the vertebral bodies. The average bone attenuation of these three vertebrae T4, T7 and T10 was calculated and expressed in Hounsfield units.

### Mortality

Deaths occurring anywhere in the United Kingdom were identified by obtaining records from the General Registry Office for Scotland. Survival time was calculated in number of days from date of CT scan until date of death with a census cut-off date of 31 December 2011.

### Statistical analysis

CAC and TAC were log transformed (normalising/linearising transformations). For CAC, subjects were classified into three pre-specificed groups: CAC = 0, 0 < CAC ≤ 400 and CAC > 400. Comparisons among the three groups were made using one way analysis of variance (ANOVA) or Kruskal-Wallis test. Univariate and multinominal logistic regression analyses were performed to estimate associations between CAC, TAC, PWV, bone attenuation and 15th percentile. Time to death was compared using Kaplan Meier curves, and Cox proportional-hazards analysis was used to estimate associations between all-cause mortality and CAC, TAC, PWV, bone attenuation and 15th percentile.

All statistical analyses were performed in SPSS version 17.0 (SPSS Inc., Chicago, IL, USA). Two-sided p-values ≤0.05 were considered statistically significant.

## Results

One hundred nineteen COPD subjects (aged 67.8 ±7.3, 66% were males, mean forced expiratory volume in 1 second [FEV_1_]% predicted was 46.0 ±17.5) were included in our analysis. Subjects with higher CAC were more likely to be older (p < 0.001), male (p = 0.03), and to have higher systolic blood pressure (p = 0.001), a history of hypertension (p = 0.002) or ischemic heart disease (p = 0.003), higher TAC (p < 0.001) and lower bone attenuation (p = 0.006) (Table 1). As expected there was a significant correlation between CAC and TAC (Spearman’s r = 0.57, p < 0.001). Higher CAC was associated with higher PWV (OR 1.62, p = 0.04) and lower bone attenuation (OR 0.32, p = 0.02), but was not associated with FEV_1_, 15th percentile and PI-950 after adjustment for age, sex and pack-years of smoking (Table 2). As described previously, PWV correlated with 15th percentile and PI-950 (r = 0.47, p < 0.001) [6]. Bone attenuation was not associated with PWV, 15th percentile or PI-950.

**Table 1:** Clinical characteristics of the total cohort and after stratification for CAC

	**Total cohort**	**CAC = 0**	**0 < CAC ≤** **400**	**CAC > 400**	**p-value**
Number of subjects	119	14	41	64	
Age, yrs	67.8 ± 7.3	58.6 ± 6.3	66.1 ± 6.2	70.8 ± 6.2	<0.001
Male, n (%)	79 (66)	7 (50)	24 (59)	48 (75)	0.03
FEV_1_, % predicted	46.0 ± 17.5	44.2 ± 13.7	43.8 ± 17.4	47.9 ± 18.3	0.47
FVC, % predicted	74.4 ± 20.6	72.6 ± 14.2	74.4 ± 21.0	74.8 ± 21.7	0.94
FEV_1_/FVC ratio	47.1 ± 12.3	46.9 ± 10.7	44.9 ± 13.5	48.6 ± 11.9	0.33
BMI, kg/m^2^	25.8 ± 5.5	25.6 ± 5.9	25.7 ± 5.2	25.9 ± 5.6	0.98
Distance walked, m	369.1 ± 112.2	406.6 ± 105.2	385.9 ± 105.0	349.8 ± 116.1	0.16
Pack-years of smoking, n	45.4 ± 20.5	44.4 ± 20.2	44.6 ± 21.7	46.2 ± 20.0	0.91
Heart rate, beats/min	74.0 ± 13.0	71.5 ± 10.1	73.4 ± 12.0	74.9 ± 14.2	0.65
Blood pressure systolic, mmHg	142.2 ± 23.9	127.7 ± 16.5	136.2 ± 20.3	149.2 ± 25.0	0.001
Blood pressure diastolic, mmHg	79.9 ± 11.3	76.6 ± 11.5	78.0 ± 9.2	81.9 ± 12.1	0.11
Hypertension, n (%)	30 (25)	0 (0)	7 (17)	23 (36)	0.002
Ischemic heart disease, n (%)	22 (19)	0 (0)	4 (10)	18 (28)	0.003
Diabetes, n (%)	7 (6)	0 (0)	2 (5)	5 (8)	0.25
TAC, median (min-max)	1540 (0–20828)	262 (0–4237)	1125 (3–8606)	3475 (0–20828)	<0.001
PWV, m/s	8.90 ± 1.63	8.35 ± 1.36	9.18 ± 1.54	8.86 ± 1.72	0.31
PI-950, median in % (min-max)	6.89 (0.12–43.2)	17.1 (0.12–39.0)	5.32 (0.12–43.2)	6.86 (0.27–38.6)	0.81
15th percentile, HU	-921.9 ± 27.8	-919.9 ± 38.3	-924.2 ± 29.7	-920.8 ± 24.1	0.82
Bone attenuation, HU	138.1 ± 35.5	162.2 ± 37.6	141.8 ± 31.9	130.4 ± 35.0	0.006

Results are presented as mean ± standard deviation unless otherwise indicated.

PWV was assessed in 104 subjects.

*BMI* body mass index, *CAC* coronary artery calcium, *FEV*_*1*_ forced expiratory volume in 1 second, *FVC* forced vital capacity, *HU* hounsfield unit, *PI* pixel index, *PWV* pulse wave velocity, *TAC* thoracic aortic calcium.

**Table 2:** Multinomial regression for CAC

	**0 < CAC** **≤** **400**		**CAC > 400**		
	**Odds ratio**	**95% CI**	**Odds ratio**	**95% CI**	**p-value**
PWV^a^	1.92	1.08 – 3.44	1.62	0.91 – 2.91	0.05
PWV^b^	2.03	1.10 – 3.74	1.62	0.87 – 3.02	0.04
Bone attenuation/SD^a^	0.46	0.21 – 1.03	0.33	0.14 – 0.77	0.02
Bone attenuation/SD^b^	0.43	0.19 – 1.00	0.32	0.13 – 0.76	0.02
FEV_1_^a^	0.82	0.22 – 3.09	0.84	0.21 – 3.29	0.96
FEV_1_^b^	0.80	0.21 – 3.05	0.82	0.21 – 3.25	0.95
15th percentile/SD^a^	0.80	0.45 – 1.45	0.64	0.45 – 1.63	0.76
15th percentile/SD^b^	0.80	0.43 – 1.48	0.84	0.43 – 1.64	0.77

Reference category CAC = 0.

^a^After adjustment for age and sex.

^b^After adjustment for age, sex and pack-years of smoking.

*CI* confidence interval, *FEV*_*1*_ forced expiratory volume in 1 second, *PWV* pulse wave velocity, *SD* standard deviation.

### Mortality

**Figure 1:**
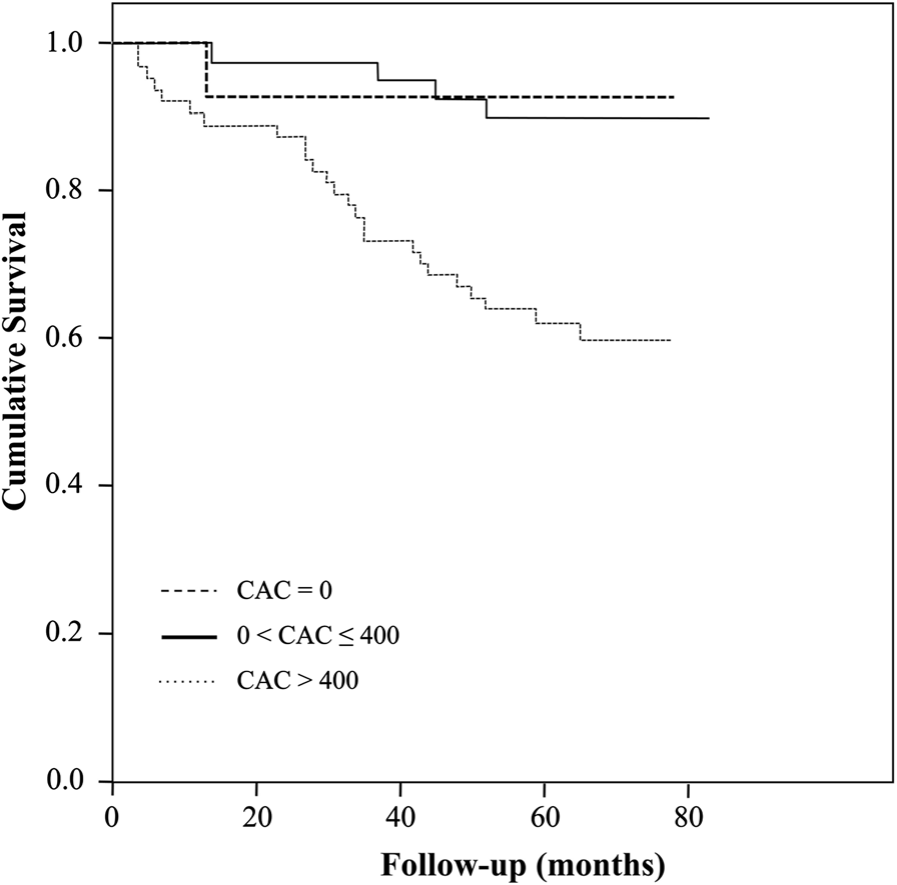
Kaplan-Meier, CAC = coronary artery calcium.

The median follow-up was 65 (0–83) months. During the observation period, 30 subjects (25%) died. A respiratory cause of death was the primary aetiology in 18 subjects (60%), and cardiovascular cause of death was the primary aetiology in 7 subjects (23%). In a Cox proportional-hazards model, CAC predicted all-cause mortality after adjustment for age, sex, FEV_1_ and pack-years of smoking (HR 2.01, 95% CI 1.13-3.58, p = 0.02, Table 3). Figure 1 and Table 4 show the Kaplan-Meier curves and numbers at risk. TAC predicted all-cause mortality similarly to CAC (Additional file 1). Lower 15th percentile (more emphysema) was associated with increased all-cause mortality after adjustment for age and sex (HR 0.65, CI 0.45-0.95, p = 0.03), but not after adjustment for age, sex, FEV_1_ and pack-years of smoking (p = 0.06) (Additional file 2). PWV and bone attenuation were not associated with all-cause mortality (Additional file 2).

**Table 3:** Cox proportional-hazards for all-cause mortality

	**CAC** **=** **0**	**0 < CAC ≤** **400**	**CAC > 400**	**Per 10-fold increase in CAC**	**p-value**
Number of subjects	14	41	64	-	-
Mortality, n (%)	1 (7)	4 (10)	25 (39)	-	-
CAC	-	1.33	6.46	2.17 (1.29 – 3.65)	0.004
CAC^a^	-	1.18	5.15	2.02 (1.13 – 3.59)	0.02
CAC^b^	-	1.14	5.09	1.94 (1.11 – 3.41)	0.02
CAC^c^	-	1.11	4.98	2.01 (1.13 – 3.58)	0.02

^a^After adjustment for age and sex.

^b^After adjustment for age, sex and FEV_1._

^c^After adjustment for age, sex, FEV_1_ and pack-years of smoking.

*CAC* coronary artery calcium, *FEV*_*1*_ forced expiratory volume in 1 second.

**Table 4:** Numbers at risk

	**CAC = 0**	**0 < CAC** **≤** **400**	**CAC > 400**
0 months	14	41	64
30 months	13	40	52
60 months	10	31	32

*CAC* coronary artery calcium.

## Discussion

We found that increased CAC was associated with increased arterial stiffness and lower bone attenuation in COPD subjects, but was not associated with extent of emphysema. CAC, TAC and extent of emphysema predicted all-cause mortality in our COPD cohort, while arterial stiffness and bone attenuation were not associated with all-cause mortality.

In line with previous studies [4,5], our data suggest a relationship between cardiovascular disease and osteoporosis in COPD patients. In our study, the association between CAC and bone attenuation was independent of age, sex and pack-years of smoking. In a prospective study in 2442 postmenopausal women, the association between osteoporosis and cardiovascular events was independent of traditional cardiovascular risk factors such as age, cigarette smoking, hypertension and hyperlipidemia [3]. These data suggest that, in addition to common risk factors, other factors might be involved in the vascular-bone relationship, including systemic inflammation [5], disturbance of the receptor activator of NF-κB/receptor activator of NF-κB ligand/osteoprotegerin system or reduced bone perfusion due to generalised atherosclerosis.

Our data showed a weak association between CAC and arterial stiffness in a COPD cohort after adjustment for age, sex and pack-years of smoking. Previous studies in older populations have found associations between CAC and arterial stiffness after adjustment for confounders [12]. Thus, in addition to common risk factors, atherosclerosis may cause arterial stiffening [13], while increased arterial stiffness in turn may promote atherosclerotic changes due to greater shear and intraluminal stresses [14].

In line with previous research [15,16], CAC was not associated with the degree of airflow obstruction or the extent of emphysema. CAC was not associated with the severity of airflow obstruction or the extent of emphysema in 1159 smokers of the Multicentre Italian Lung Detection (MILD) study [16], and CAC and proximal aortic calcification were not associated with the severity of airflow obstruction in subjects without clinical cardiovascular disease of the Multi-Ethnic Study of Atherosclerosis (MESA) study [15,17]. Notably, as reported previously in this cohort, arterial stiffness did correlate with the extent of emphysema in COPD subjects [6]. The relationship between arterial stiffness and emphysema might be due to connective tissue degradation [6] or elastin degradation [18].

Bone attenuation was not associated with the extent of emphysema. In contrast, a recent review stated that more severe emphysema is associated with reduced bone mineral density [19]. Bon and colleagues [7] showed that the extent of emphysema was independently associated with bone mineral density in 190 current and former smokers, and Ohara and colleagues [20] demonstrated that the extent of emphysema was independently associated with CT measured bone density of the thoracic and lumbar vertebrae in 65 male COPD subjects. The discrepancy between our data and their data might be due to differences in the study population (e.g. smokers versus COPD patients) and methods used (e.g. attenuation threshold of low attenuation areas).

CAC and TAC predicted all-cause mortality after adjustment for age, sex, FEV_1_ and pack-years of smoking. Several studies have shown that CAC is a predictor of cardiovascular events and mortality in smokers [21,22]. Here we show that CAC was a strong predictor of all-cause mortality in a COPD cohort. The hazard ratios reported in our COPD subjects were comparable with those reported in smokers and subjects without known coronary artery diseases. In 1159 smokers, the hazard ratio of CAC > 400 was 4.00 (CI 1.17-13.7) for all-cause mortality compared with CAC ≤ 400 after adjustment for age, sex, body mass index and history of diabetes or hypertension [16]. In our study, the hazard ratio of CAC > 400 was 4.6 (CI 1.63-13.12, p = 0.004) compared with CAC ≤ 400 after adjustment for age, sex, FEV_1_ and pack-years of smoking. In 3966 subjects without known coronary artery diseases, the crude hazard ratio of CAC ≥ 400 was HR 6.54 (CI 3.51-12.21) for all-cause mortality [23], and in our study the crude hazard ratio of CAC > 400 was 6.46.

Although CAC and TAC were associated with all-cause mortality, arterial stiffness was not associated with all-cause mortality in our COPD cohort. This finding is in contrast to previous studies that have demonstrated a relationship between increased arterial stiffness and all-cause mortality in subjects with chronic kidney disease [24,25]. The lack of a relationship between increased arterial stiffness and mortality in our study might be explained by the fact that we measured carotid-radial PWV rather than carotid-femoral PWV which is the gold standard [26]. A study in 305 end-stage renal disease patients showed indeed that carotid-femoral PWV was an independent predictor of cardiovascular mortality, whereas carotid-radial and femoral-posterior tibial PWV were not associated with mortality [27].

In addition, the extent of emphysema was associated with all-cause mortality after adjustment for age and sex, but not after adjustment for age, sex and FEV_1_. As such, it may be that emphysema causes increased mortality indirectly through reducing lung function. Our findings are in line with previous studies that have shown associations between the extent of emphysema and all-cause mortality in COPD subjects [28].

Although lower bone attenuation was associated with increased CAC, bone attenuation was not associated with all-cause mortality in our COPD cohort. In contrast, previous studies have shown that bone density and rate of bone loss are related to mortality in elderly men and women [29,30]. The Rotterdam Study demonstrated that in elderly men the risk of mortality increased when bone density was below average, and that the relationship between bone density and mortality was nonlinear [29]. In addition, Szulc and colleagues [30] showed in 781 elderly men that bone resorption markers predicted mortality more strongly than bone mineral density. The lack of a relationship between bone attenuation and mortality in our study might be due to the study population (e.g. COPD subjects rather than elderly subjects) and the methods used (e.g. bone attenuation of the thoracic vertebrae rather than bone mineral density of the hip, lumbar spine or whole body).

Our study has several limitations. First, bone attenuation was assessed on low-dose chest CT while dual energy X-ray absorptiometry is the gold standard. However, previous data have demonstrated that bone attenuation measured on CT is strongly correlated with bone mineral density assessed on dual energy X-ray absorptiometry [11]. Second, CAC and TAC were measured on non-gated scans, however, it has been shown previously that measurements of CAC and TAC on non-gated chest CT scans show good agreement with measurements from electrocardiographically gated chest CT scans [9]. Third, arterial stiffness was measured as carotid-radial PWV rather than carotid-femoral PWV which is the gold standard [26]. Although our study has several limitations, we measured several COPD related manifestations such as CAC, TAC, bone attenuation and extent of emphysema in a well-characterised COPD cohort.

Our data showed that CAC, TAC and emphysema which were measured on low-dose chest CT predicted all-cause mortality in COPD subjects. We suggest that low-dose chest CT might contribute to early diagnosis of COPD related manifestations and improved risk stratification, although further evidence, ideally from randomised controlled trials, are required to determine the benefits of this screening approach in COPD patients.

## Conclusions

Our study showed that increased CAC was associated with increased arterial stiffness and lower bone attenuation in COPD subjects. CAC, TAC and emphysema predicted all-cause mortality in our COPD cohort, suggesting that quantitative assessment of CAC, TAC and emphysema on chest CT provides relevant prognostic information in COPD subjects.

## Abbreviations

BMI: Body mass index; CAC: Coronary artery calcification; CI: Confidence interval; COPD: Chronic obstructive pulmonary disease; CT: Computed tomography; DEXA: Dual energy x-ray absorptiometry; FEV1: Forced expiratory volume in 1 second; FVC: Forced vital capacity; HR: Hazard ratio; HU: Hounsfield unit; OR: Odds ratio; PI: Pixel index; PWV: Pulse wave velocity; SD: Standard deviation; TAC: Thoracic aortic calcification.

## Competing interest

Elisabeth A.P.M. Romme was the recipient of a European Respiratory Society Fellowship (STRTF 381–2011).

## Authors’ contributions

EAR contributed to the study idea and design, analysis and interpretation of the results and drafting of the manuscript. DM contributed to the study idea and design, analysis and interpretation of the results, and drafting of the manuscript. JM contributed to the study idea and design, analysis and interpretation of the results, and revision of the manuscript. EvB contributed to the analysis and interpretation of the results, and revision of the manuscript. CP contributed to the analysis and interpretation of the results, and revision of the manuscript. EPR contributed to the analysis and interpretation of the results, and revision of the manuscript. FS contributed to the analysis and interpretation of the results, and revision of the manuscript. EW contributed to the analysis and interpretation of the results, and revision of the manuscript. WM contributed to the study idea and design, analysis and interpretation of the results, and revision of the manuscript. All authors read and approved the final manuscript.

## Supplementary Material

Additional file 1 Cox proportional-hazards for all-cause mortality. Click here for file

Additional file 2 Cox proportional-hazards for all-cause mortality. Click here for file
