# Supplementary material for: Associations between COPD related manifestations: a cross-sectional study
Source: Respir Res. 2013 Nov 19;14(1):129. doi: 10.1186/1465-9921-14-129 (PMC3840707; doi:10.1186/1465-9921-14-129)
Supplement: Additional file 1 — Cox proportional-hazards for all-cause mortality. [file 1465-9921-14-129-S1.pdf]

Additional file 1: Cox proportional-hazards for all-cause mortality

	Per 10-fold increase in TAC	95% CI	p-value
TAC	2.20	1.20 - 4.03	0.01
TAC [a]	2.08	0.99 - 4.38	0.06
TAC [b]	1.97	0.93 - 4.19	0.08
TAC [c]	2.09	0.95 - 4.59	0.07

[a] after adjustment for age and sex

[b] after adjustment for age, sex and FEV_1

[c] after adjustment for age, sex, FEV_1 and pack-years of smoking

CI=confidence interval, FEV_1=forced expiratory volume in 1 second, TAC=thoracic aortic calcification
