# Supplementary material for: Associations between COPD related manifestations: a cross-sectional study
Source: Respir Res. 2013 Nov 19;14(1):129. doi: 10.1186/1465-9921-14-129 (PMC3840707; doi:10.1186/1465-9921-14-129)
Supplement: Additional file 2 — Cox proportional-hazards for all-cause mortality. [file 1465-9921-14-129-S2.pdf]

Additional file 2: Cox proportional-hazards for all-cause mortality

	Hazard ratio	95% CI	p-value
PWV	0.91	0.72 - 1.16	0.44
PWV [a]	0.86	0.67 - 1.10	0.23
PWV [b]	0.85	0.67 - 1.08	0.18
PWV [c]	0.81	0.62 - 1.06	0.13
Bone attenuation/ SD	0.98	0.68 - 1.40	0.90
Bone attenuation/ SD [a]	1.04	0.72 - 1.50	0.84
Bone attenuation/ SD [b]	1.10	0.76 - 1.57	0.62
Bone attenuation/ SD [c]	1.11	0.77 - 1.60	0.58
15th percentile/ SD	0.70	0.49 - 1.00	0.05
15th percentile/ SD [a]	0.65	0.45 - 0.95	0.03
15th percentile/ SD [b]	0.69	0.46 - 1.04	0.07
15th percentile/ SD [c]	0.66	0.43 - 1.01	0.06

[a] after adjustment for age and sex

[b] after adjustment for age, sex and FEV_1

[c] after adjustment for age, sex, FEV_1 and pack-years of smoking

CI=confidence interval, FEV_1=forced expiratory volume in 1 second, PWV=pulse wave velocity, SD=standard deviation
